# Human T Lymphotropic Virus Type 1 protein Tax reduces histone levels

**DOI:** 10.1186/1742-4690-5-9

**Published:** 2008-01-31

**Authors:** James M Bogenberger, Paul J Laybourn

**Affiliations:** 1 Department of Biochemistry and Molecular Biology, Colorado State University, Fort Collins, Colorado, USA

## Abstract

**Background:**

Human T-Lymphotropic Virus Type-1 (HTLV-1) is an oncogenic retrovirus that causes adult T-cell leukemia/lymphoma (ATLL). The virally encoded Tax protein is thought to be necessary and sufficient for T-cell leukemogenesis. Tax promotes inappropriate cellular proliferation, represses multiple DNA repair mechanisms, deregulates cell cycle checkpoints, and induces genomic instability. All of these Tax effects are thought to cooperate in the development of ATLL.

**Results:**

In this study, we demonstrate that histone protein levels are reduced in HTLV-1 infected T-cell lines (HuT102, SLB-1 and C81) relative to uninfected T-cell lines (CEM, Jurkat and Molt4), while the relative amount of DNA per haploid complement is unaffected. In addition, we show that replication-dependent core and linker histone transcript levels are reduced in HTLV-1 infected T-cell lines. Furthermore, we show that Tax expression in Jurkat cells is sufficient for reduction of replication-dependent histone transcript levels.

**Conclusion:**

These results demonstrate that Tax disrupts the proper regulation of replication-dependent histone gene expression. Further, our findings suggest that HTLV-1 infection uncouples replication-dependent histone gene expression and DNA replication, allowing the depletion of histone proteins with cell division. Histone proteins are involved in the regulation of all metabolic processes involving DNA including transcription, replication, repair and recombination. This study provides a previously unidentified mechanism by which Tax may directly induce chromosomal instability and deregulate gene expression through reduced histone levels.

## Background

Human T-lymphotropic virus type 1 (HTLV-1) is a complex retrovirus originally isolated in 1980 from a patient with cutaneous T-cell lymphoma [1]. After the identification of HTLV-1 as the first human retrovirus, it was demonstrated to be associated with a malignancy of T lymphocytes known as adult T-cell leukemia/lymphoma (ATLL) [2-6]. ATLL is now known to be a direct result of HTLV-1 infection [7]. HTLV-1 is also associated with non-malignant, lymphocyte-mediated inflammatory diseases, including the neurodegenerative disease tropical spastic paraparesis/HTLV-I associated myelopathy (TSP/HAM) [8-10].

The pathogenesis of HTLV-1 differs from that of other known retroviruses. Unlike the acutely transforming retroviruses, HTLV-1 does not encode an oncogene transduced from a host genome [11]. Additionally, HTLV-1 can induce ATLL independently of the cis-acting effects of proviral integration, distinguishing it from the slowly transforming, cis-acting retroviruses [12]. Like other retroviruses, the HTLV-1 genome consists of two long-terminal repeats (LTRs) flanking the common retroviral genes *gag, pro, pol*, and *env *[11]. HTLV-1 contains an additional genomic segment between the *env *gene and the 3' LTR, called the pX region [13]. The pX region contains four partially overlapping open reading frames (ORFs), encoding several nonstructural or accessory viral gene products required for viral replication and infectivity [14-19]. ORF IV encodes the viral transcription factor Tax [13]. Tax is essential for replication of the HTLV-1 genome and is required for HTLV-1 pathogenesis. Although there is no known cellular homolog, Tax is considered to be an oncoprotein. Tax has been shown to be necessary and sufficient to transform primary T-cells and form tumors in transgenic mice [6,20-24].

The oncogenic capacity of Tax resides in its ability to induce inappropriate cell proliferation, inhibit DNA repair pathways, deregulate cell cycle checkpoint controls, and induce genomic instability. These effects of Tax on cellular homeostasis are mediated both by Tax deregulation of cellular gene expression and by direct Tax interactions with multiple regulators of cellular homeostasis [25-27]. It is thought that all of these effects of Tax function to promote viral replication [28]. HTLV-1 infected cells produce virtually no cell-free infectious virus particles. Rather, infection appears to be mediated by cell-to-cell contact [29-31]. The ability of Tax to drive cell cycle progression and inactivate cell cycle checkpoints promotes replication of the proviral genome, but consequently results in an increased frequency of neoplasia in the host cell.

In addition to Tax, the HTLV-1 genome encodes five other accessory proteins, p12^I^, p30^II^, p13^II^, HBZ and Rex. Of these proteins, p12^I^, p30^II ^and p13^II ^are not essential for viral replication in vitro and Rex is not required for T-cell immortalization [32]. However, experiments in animal models using infectious viral clones suggest that these other accessory proteins are important for productive infection in vivo [33-35]. Accessory protein p12^I ^localizes to the endoplasmic reticulum and Golgi and appears to promote cell survival and proliferation through increased cytoplasmic Ca^++ ^levels [36]. This protein may also participate in immune response evasion through reduction of MHC expression [37]. Similarly, p30^II ^may function to promote cell proliferation at the same time as viral latency through effects on CBP/p300 function, as well as, Tax and Rex mRNA translation [38-40]. Accessory protein p13^II ^localizes to the mitochondrial inner membrane and affects K^+ ^permeability and Ca^++ ^uptake suggesting a function through Ca^++ ^signaling, as well [41]. The HBZ open reading frame is transcribed from the minus DNA strand using a promoter in the 3'-LTR and is thought to produce both a protein and an RNA product [42-44]. HBZ RNA appears to promote T-cell proliferation while the protein product suppresses viral transcriptional activation by Tax [45,46]. Rex function is important for viral replication, as it drives the transition to production of full-length genomic RNA [47,48]. Rex is thought to recognize a sequence in the viral RNA (Rex response element; RxRE), repress splicing, and promote nuclear export [49]. Rex has also been shown to be dispensable for virion production in vitro, but to be essential in vivo [50].

Evidence from clinical studies suggests that fewer than 5% of infected individuals develop ATLL and that manifestation of ATLL occurs after a long latent period between 20–60 years [51]. These observations suggest that secondary events are required in the progression from HTLV-1 infection to T-cell transformation and the development of ATLL. Multi-step oncogenesis is thought to be driven by reduced DNA repair capacity and/or genomic instability [52].

Both ATLL cells and those immortalized by HTLV-1 infection and Tax expression in culture exhibit a broad spectrum of chromosomal abnormalities, including deletions, duplications, translocations and anneuploidy [53,54]. Tax expression and DNA damage have been correlated in studies demonstrating that Tax expression results in the formation of micronuclei [55,56]. Tax expression has been shown to induce gene amplification, as well [57]. Tax expression has been observed to cause an uncoupling of DNA synthesis from cell division and result in the formation of multinucleated giant cells exhibiting decondensed, highly convoluted and polylobulated nuclei [58]. This phenotype is similar to that of the large lymphocytes with cleaved or cerebriform nuclei observed at higher frequencies in HTLV-1 positive individuals [59]. The clastogenic effects of Tax have been correlated with unstabilized double strand DNA breaks [60]. Currently, there is no clear mechanism for how chromosomal abnormalities arise in HTLV-1 infected cells and there is no evidence linking a specific chromosomal abnormality to the development of ATLL.

Histones are critical components of chromatin and are required for the proper packaging of genomic DNA within the eukaryotic nucleus. The core histones assemble with DNA into nucleosome core particles, the fundamental units of chromatin. Arrays of nucleosome core particles constitute a chromatin fiber, the primary level of chromatin structure. The chromatin fiber is further compacted through the formation of additional levels of higher order chromatin structure. Linker histones are thought to modulate nucleosome dynamics and higher-order chromatin structure formation. Ultimately, the chromatin fiber is compacted by non-histone proteins forming metaphase chromatids or chromosomes, the most compacted state of genomic DNA. Histones and chromatin structure play a central role in the regulation of all DNA metabolic processes, including transcription, repair, replication and recombination [61].

In the present study we demonstrate that HTLV-1 infected T-cell lines exhibit a reduction of replication-dependent histone protein and transcript levels relative to uninfected T-cell lines. However, we detect no concomitant decrease in genomic DNA. Further, we show that Tax expression in an uninfected T-cell line is sufficient to repress replication-dependent histone transcript levels. These results provide a previously unidentified mechanism through which Tax may deregulate gene expression. Additionally, these results provide a mechanism by which Tax may *directly *induce genomic instability.

## Results

Phenotypically, ATLL cells exhibit enlarged, polylobulated nuclei as well as micronuclei [56,59]. In addition, the viral transcription factor Tax is thought to deregulate the expression of hundreds of cellular genes [62-65]. We were interested in investigating the possibility that these attributes result, in part, from defects in forming proper chromatin structure. Since histones are critical to the formation of chromatin structure, we hypothesized that HTLV-1 infection and Tax perturb normal histone gene expression.

### Histone H3 and H1 levels are reduced in HTLV-1 infected T-cell lines

To quantify histone protein levels we sought to measure histone protein and DNA content simultaneously using a flow cytometry based assay. This assay has several advantages over other methods of measuring cellular histone protein levels. In normal cells, histone protein levels are dependent on cellular DNA content. This fact has important consequences for the measurement of histone protein levels in different cell lines. Differences in the amount of DNA per haploid complement will result in concomitant differences in histone protein levels during interphase. Additionally, as cells replicate genomic DNA during S-phase, histone protein levels will increase along with the DNA content. Thus, simultaneous quantification of histone protein and DNA content allowed us to normalize histone protein levels to DNA content. The ability to detect the slight increases in both histone protein and DNA content as cells progress through S-phase attests to the quantitative sensitivity of our assay.

Histone H3 and DNA content were measured simultaneously by flow cytometry. The antibody used (see Materials and Methods) is directed against the H3 carboxyl-terminus and recognizes modified forms of H3 and H3 variants. In addition, H3 forms the core heterotetramer of nucleosomes, along with H4. It is important to note that somatic cells, regardless of cell cycle phase, contain no significant histone pools and levels of all four core histones and total linker histone are finely balanced. It has been estimated that even during S phase, the soluble histone pool represents about 0.1% of the total histone in human cells [66,67]. Therefore, the levels of H3 measured using this antibody are indicative of the nucleosomal levels on the chromosomes. During flow cytometry, the level of anti-H3 antibody bound per cell was measured using an Alexa 488-conjugated secondary antibody. Cells were also stained with propidium iodide (PI) to determine genomic DNA content. Unstained cells, as well as cells treated with primary antibody only, secondary antibody only, PI only, or primary and secondary antibody only were included as controls in every experimental analysis to determine background fluoresence and in instrument calibration.

**Figure 1:**
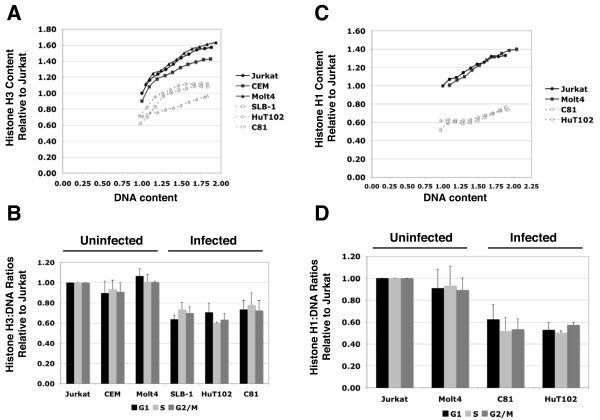
**Histone H3 and H1 levels are reduced relative to genomic DNA content**. Histone H3 and genomic DNA content were measured simultaneously in uninfected T-cell lines (CEM, Jurkat and Molt4) and HTLV-1 infected T-cell lines (SLB-1, HuT102 and C81) using flow cytometry. H3 levels were measured using a primary antibody directed against the C-terminal domain and an Alexa-488 labeled secondary antibody. DNA content was measured using propidium iodide. **(A) **Mean histone H3 immunofluorescent intensity was traced from G1 through G2/M by defining a narrow window on the DNA histogram and plotted against the genomic DNA content for the corresponding cell population defined by the window. **(B) **The average histone H3:DNA ratios of three experiments during G1, Mid-S and G2/M are shown for HTLV-1 infected and uninfected T-cell lines (see Table 1 for p-values) **(C) **Histone H1 and genomic DNA content were measured simultaneously in uninfected T-cell lines (Jurkat and Molt4) and in HTLV-1 infected T-cell lines (C81 and HuT102) using flow cytometry. H1 levels were measured using a primary antibody directed against the C-terminal domain and an Alexa-488 labeled secondary antibody. DNA content was measured using propidium iodide. Mean histone H1 immunofluorescent intensity was traced from G1 through G2/M by defining a narrow window on the DNA histogram and plotted against the genomic DNA content for the corresponding cell population defined by the window. **(D)** The average histone H1:DNA ratios during G1, Mid-S and G2/M are shown for HTLV-1 infected and uninfected T-cell lines (see Table 2 for p-values).

Mean histone H3 signal intensity was traced from G1 through G2/M and plotted against mean genomic DNA content (Figure 1A). This was accomplished by defining a narrow window on each DNA histogram so that the histone H3 levels for the corresponding cell population defined by the window could be determined at a particular DNA content level. Our results show that upon initiation of DNA replication at the onset of S phase, cells exhibit a near linear increase in both DNA and histone H3 content. As cells approach G2/M histone H3 immunofluoresence was observed to plateau in all T-cell lines examined (both infected and uninfected), while PI fluorescence (DNA content) continued to increase in a near linear fashion. The plateau in H3 immunofluoresence observed may be explained by a decrease in H3 antibody epitope accessibility during chromosome condensation. Nevertheless, the simultaneous measurement of histone levels and DNA content using flow cytometry is clearly sensitive and quantitative enough to accurately measure the slight increases in both DNA and histone associated with genome replication as cells progress through S phase of the cell cycle. To display the histone H3 levels measured for each of the cell lines examined, the average histone H3:DNA ratios corresponding to G1, Mid-S and G2/M cell populations were calculated for each cell line and expressed relative to Jurkat (Figure 1B).

To summarize our results, histone H3 levels are not significantly different between each of the three uninfected T-cell lines, Jurkat, CEM and Molt4. In clear contrast, H3 levels are reduced 20 to 40% in the HTLV-1 infected T-cell lines, SLB-1, HuT102 and C81, relative to Jurkat cells. The p-values associated with the measurement of histone H3:DNA ratios are shown in Table 1. The reduction in histone H3 observed is consistent throughout the cell cycle.

**Table 1:** P-values for data in Figure 1B. Note: Jurkat values have been set to 1.0.

**H3:DNA**	**G1**	**Mid-S**	**G2/M**
**CEM**	1.7 × 10^-1^	2.2 × 10^-1^	1.5 × 10^-1^
**Molt4**	1.6 × 10^-1^	4.2 × 10^-1^	2.6 × 10^-1^
**SLB-1**	3.0 × 10^-3^	1.5 × 10^-2^	1.1 × 10^-2^
**HuT102**	2.2 × 10^-2^	9.4 × 10^-5^	6.4 × 10^-3^
**C81**	2.6 × 10^-2^	6.0 × 10^-2^	3.3 × 10^-2^

Histone H1 protein levels were determined using the same flow cytometry-based assay. Histone H1 levels are plotted against genomic DNA content (Figure 1C) and histone H1:DNA ratios are determined (Figure 1D). Histone H1 protein levels are not significantly different in the two uninfected T-cell lines examined, Jurkat and Molt4. However, histone H1 protein levels are reduced by 40% in the two infected T-cell lines examined, C81 and HuT102 as compared to the uninfected T-cell lines, Molt4 and Jurkat. The p-values associated with the measurement of histone H1:DNA ratios are shown in Table 2. The reduction of histone H1 is also consistent throughout the cell cycle.

**Table 2:** P-values for data in Figure 1D. Note: Jurkat values have been set to 1.0.

**H1:DNA**	**G1**	**Mid-S**	**G2/M**
**Molt4**	3.5 × 10^-1^	3.9 × 10^-1^	2.5 × 10^-1^
**C81**	1.1 × 10^-1^	8.0 × 10^-2^	6.0 × 10^-2^
**HuT102**	5.0 × 10^-2^	1.0 × 10^-2^	2.0 × 10^-2^

Having unequivocally determined that histone protein levels are reduced in HTLV-1 infected T-cell lines, we proceeded to determine if this was a result of reduced histone message levels.

### Replication-dependent histone transcript levels are reduced in HTLV-1 infected T-cell lines

**Figure 2:**
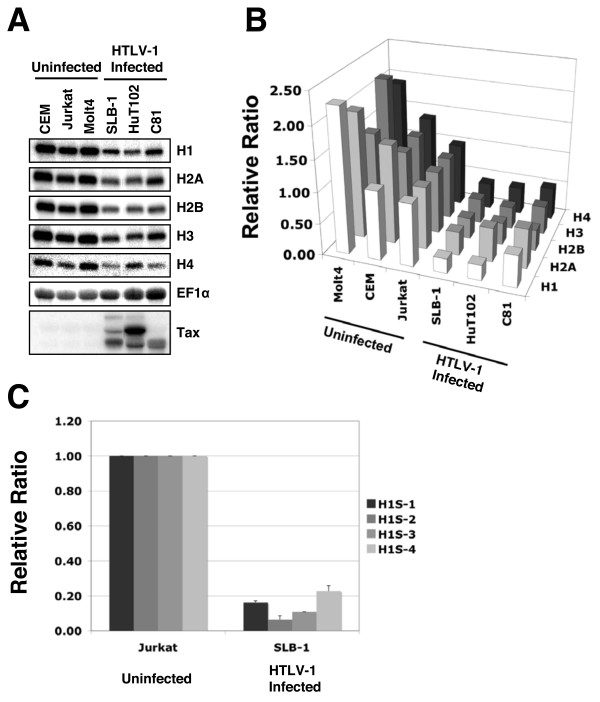
**Replication-dependent core and linker histone transcripts are reduced in HTLV-1 infected T-cell lines**. **(A) **Core and linker histone transcript levels were measured in uninfected T-cell lines (CEM, Jurkat and Molt4) and HTLV-1 infected T-cell lines (SLB-1, HuT102 and C81) by northern blotting. **(B) **Graphical representation of northern blot quantification. Histone transcript signals were normalized to EF1α transcript signals. Values for all T-cell lines are expressed relative to Jurkat cells. P-values associated with northern blot measurements of total histone transcripts for each cell line were: CEM – 2.3 × 10^-3^, Molt4 – 1.1 × 10^-4^, SLB-1 – 5.8 × 10^-12^, HuT102 – 2.9 × 10^-8^, C81 – 2.8 × 10^-6 ^**(C) **Linker histone isoform transcripts are reduced in HTLV-1 infected T-cell lines. Somatic linker histone isoform transcript levels were measured in an uninfected T-cell line (Jurkat) and an HTLV-1 infected T-cell line (SLB-1), using reverse-transcription and real-time PCR. Linker histone transcript levels were normalized to EF1α transcript levels and values were expressed relative to transcript levels in Jurkat cells [110].

The replication-dependent histones are expressed from multiple gene copies. While their amino acid sequences are highly conserved they are divergent enough in their DNA sequences as to require a separate primer set for the measurement of transcript from each of the gene copies by real-time PCR. Therefore, Northern blot analysis was employed allowing the measurement of total transcript levels for each replication-dependent histone type. Histone transcript levels in each cell line were normalized against EF1α. Northern blot analyses revealed a reduction of histone transcripts from all of the replication-dependent histone genes (Figure 2A). Quantification of the Northern blot results revealed a reduction in core and linker histone transcript levels ranging from two to five-fold, depending on the histone, in HTLV-1 infected T-cell lines (HUT102, SLB-1 and C81) as compared to Jurkat T-cells (Figure 2B). On average, histone transcripts are reduced by 66%, 61% and 50% in SLB-1, HuT102 and C81 cells respectively. Histone transcript levels in the other uninfected T-cell lines examined, Molt4 and CEM, were either not significantly different from or slightly higher than Jurkat. These results indicate that HTLV-1 infection is reducing histone protein levels through an effect on histone transcript levels.

### All linker histone isoform transcript levels are reduced in HTLV-1 infected T-cell lines

We used reverse-transcription and real-time PCR to determine the transcript levels of each of the somatic linker histone isoforms. Real-time PCR quantification of linker histone cDNAs generated from an uninfected T-cell line (Jurkat) and from an HTLV-1 infected T-cell line (SLB-1) reveals a significant reduction of somatic linker histone transcripts in the HTLV-1 infected T-cell line when compared to the uninfected T-cell line (Figure 2C). H1^S^-1, H1^S^-2, H1^S^-3, and H1^S^-4 transcripts are 6, 14, 9 and 4-fold lower in SLB-1 cells, respectively, relative to Jurkat T-cells. These results indicate that HTLV-1 infection is reducing histone protein levels through an effect on histone transcript levels.

### Tax expression alone reduces histone transcript levels in an uninfected T-cell line

The HTLV-1 provirus encodes several viral proteins, but only the Tax protein is known to be oncogenic. To determine if Tax alone is sufficient to repress histone gene expression, we transiently transfected an uninfected T-cell line (Jurkat) with a Tax expression vector (pSG-Tax) or empty vector (pUC-19). Both sets of cells were cotransfected with a vector encoding a surface molecule (truncated mouse MHC Class I) to allow selection of transfectants by their retention on magnetic beads conjugated to antibody recognizing this unique surface marker. Real-time PCR was used to evaluate all of the somatic linker histone mRNAs and representative family members of the core histone genes in Tax expressing and pUC-19 transfected Jurkat cells. Tax expression was verified by both western blot and real-time PCR (not shown).

**Figure 3:**
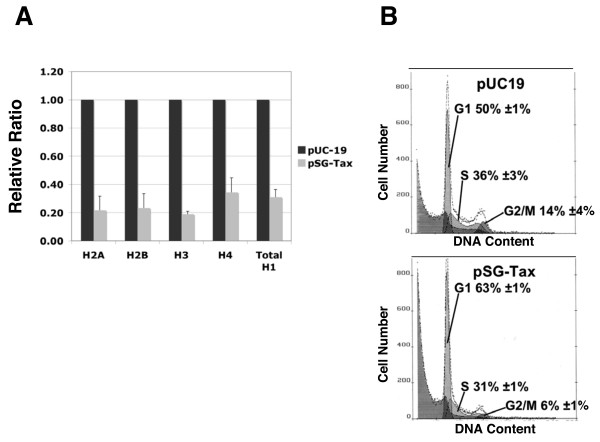
**Tax expression is sufficient for reduction of replication-dependent histone transcript levels**. **(A) **Histone transcript levels were measured in pSG-Tax transfected Jurkat cells and empty vector transfected Jurkat cells using reverse-transcription and real-time PCR. P-values associated with the measurement of histone transcripts in pUC-19 vs pSG-Tax transfected Jurkat cells were: H2A – 3.7 × 10^-6^, H2B – 3.4 × 10^-5^, H3 – 5.6 × 10^-3^, H4 – 3.5 × 10^-2^, average H1 – 1.1 × 10^-2^. **(B) **DNA content analysis reveals similar cell cycle distributions for pSG-Tax transfected and empty vector transfected Jurkat cells.

Both core and linker histone mRNAs were reduced in Tax expressing Jurkat cells as compared to pUC-19 transfected Jurkat cells (Figure 3A). Core and linker histone mRNA levels are reduced in the range of three- to five-fold, similar to the reduction observed in HTLV-1 infected T-cell lines. Replication-dependent histones are only transcribed in S phase of the cell cycle. To eliminate the possibility that Tax expressing cells reduce replication-dependent histone transcript levels by reducing the proportion of cells in S phase, we determined cell cycle distributions of pSG-Tax and pUC-19 transfected Jurkat cells. pSG-Tax and pUC-19 transfected Jurkat cells used for RNA extraction were subjected to cell cycle analysis. The percentage of cells in S phase of Tax expressing Jurkat cell populations was 31%, while that of pUC-19 transfected Jurkat cells was 36% (Figure 3B). The reduction of histone mRNAs observed in Tax expressing Jurkat cells due to the lower proportion of cells in S phase is no more than 14% (31/36). Therefore, Tax effects on cell cycle distribution do not account for the remaining 52 to 66% reduction in histone transcript levels observed in these Tax transfection studies.

## Discussion

In this study we have shown that histone protein levels are reduced 20 to 40% in HTLV-1 infected T-cell lines as compared to uninfected T-cell lines and that this reduction correlates with decreased histone transcript levels. Additionally, we have shown that Tax alone is sufficient to decrease histone transcript levels. Replication-dependent histones are expressed only in S phase, so it was formally possible that the reduction in histone transcript levels observed after Tax expression in an uninfected T-cell line was the result of a significant decrease in the proportion of the cell population in S phase. We have ruled out this possibility by analyzing cell cycle distributions of Tax expressing T-cells as compared to empty vector transfected T-cells (Figure 3B). Further, by measuring histone H3 levels and DNA content per cell simultaneously through flow cytometry we have demonstrated that the reduced histone levels observed in HTLV-1 infected T-cell lines do not occur through a reduction in the amount of genomic DNA in these cells. Rather, our results indicate that Tax deregulates histone production and this occurs, at least in part, through a decrease in histone gene transcripts resulting in reduced histone proteins assembled into nucleosomes on the genomic DNA. Furthermore, our data indicate that HTLV-1 infection can uncouple replication-dependent histone gene expression from DNA synthesis. It is important to point out that we cannot exclude cooperative effects from one or more of the other accessory HTLV-1 proteins in either the reduction of histone levels or the uncoupling of histone expression from DNA replication. However, Tax is sufficient to reduce histone mRNAs.

### Replication-dependent histone genes

In normal somatic cells, expression of the majority of histone genes is tightly coupled to the rate of DNA replication during S phase of the cell cycle. Thus, they are termed replication-dependent histone genes. Under normal circumstances inhibition of histone synthesis results in DNA replication arrest. Conversely, blocking DNA replication results in a coordinate inhibition of histone expression [68]. The coordination of histone expression and DNA synthesis is critical in the maintenance of chromosomal integrity [69]. Histone chaperones and chromatin assembly factors normally cooperate in the deposition of histones onto nascent DNA as it emerges from the replication fork [70]. The down-regulation of histone gene expression in response to decreased rates of DNA synthesis prevents the accumulation of excess histones, which has been shown to be highly cytotoxic and to severely compromise the transmission of chromosomal information [71]. Since there is no significant pool of non-nucleosomal histones, chromatin assembly at the replication fork is dependent on de novo histone expression.

Mammalian cell division requires the synthesis of 10^8 ^histone proteins during the brief S phase. In order to meet these demands, cells contain multiple copies of the replication-dependent histone genes. Genomic sequence analysis has revealed 59 replication-dependent core histone genes and 6 replication-dependent linker histone genes, clustered at 4 different chromosomal loci in human cells [72]. The expression of all the replication-dependent histone genes is coordinately regulated during S phase. This coordinate regulation is mediated through both transcriptional and post-transcriptional mechanisms. It is not known how each of these different transcriptional and post-transcriptional mechanisms of histone gene regulation are coupled to the rate of DNA synthesis in proliferating cells.

### Replication-dependent histone gene transcriptional regulation

The rate of transcription of all the replication-dependent histone genes increases three- to five-fold as cells enter S phase. Studies have shown that proper cell cycle-dependent transcriptional regulation of each histone subtype involves unique cis-regulatory elements and unique transcription factors for each histone subtype. It is not known how these different DNA-binding transcription factors are coordinated to activate transcription of all the replication-dependent histone genes in response to cell cycle signaling [73,74]. Recently, non DNA-binding proteins have been identified that may be involved in coordinating histone gene transcription. Hir1p and Hir2p are cell cycle regulators thought to be responsible for transcriptional co-repression of histone gene expression in *Saccharomyces cerevisiae *[75]. HIRA is the mammalian homolog of these yeast proteins and may regulate histone gene expression in mammalian cells. HIRA has been shown to display histone chaperone function independent of DNA synthesis [76]. It has also been shown to be phosphorylated by cyclin E/cdk2 during S phase and is thought to be inhibited by this phosphorylation. The ectopic expression of HIRA is known to result in S phase arrest and reduced histone transcript levels, suggesting a negative function for HIRA in replication-dependent histone gene expression outside of S phase [77].

NPAT is another mammalian protein thought to function in the coordinate expression of replication-dependent histone genes [78,79]. NPAT has also been shown to be an in vivo substrate of cyclin E/cdk2. Phosphorylation of NPAT correlates with the colocalization of NPAT to histone processing bodies. Inhibition of NPAT gene expression impedes cell cycle progression and decreases histone gene expression in mammalian cells [80]. Thus, NPAT is thought to participate in the up-regulation of replication-dependent histone gene expression during S phase.

### Replication-dependent histone gene post-transcriptional regulation

Post-transcriptional mechanisms of histone gene expression are responsible for an additional eight- to ten-fold increase in mRNA levels as cells enter S phase [81,82]. The replication-dependent histones do not contain introns and are not polyadenylated. Instead, they all contain a very similar 3' stem-loop structure bound by a conserved protein in mammals, stem-loop binding protein (SLBP) [83,84]. SLBP mediates the only RNA processing event required for production of mature histone mRNAs, a 3'-endonucleolytic cleavage [85]. In addition, SLBP binding regulates the half-life and translational efficiency of histone mRNAs [86-88].

### Potential mechanisms of Tax deregulation of histone expression

As the promoters for the genes encoding the four replication-dependent histones have been shown to utilize unique cis-acting elements bound by different sequence-specific trans-acting factors during S-phase induced expression, Tax mediated repression of histone expression is likely to occur through an effect on one or more of the coordinating regulators of histone expression, HIRA, NPAT, or SLBP. The difference in reduction of histone protein levels (20 to 40%) and histone transcript levels (50 to 80%) suggests some compensation may occur through increased mRNA stability, translation efficiency or protein stability. In addition, results from this study suggest that HTLV-1 infection is able to uncouple the interdependence of histone gene expression and DNA synthesis. There is little known about the mechanism coupling histone synthesis and DNA replication. One candidate for this function is the histone chaperone Asf1 (Asf1a and Asf1b in mammalian cells), which has been shown to regulate histone levels during replicational stress [89]. Tax may provide a useful probe to investigate this coupling mechanism, as well as the mechanism of coordinate histone gene regulation in general.

### Potential effects of histone level reduction

We find HTLV-1 infected cells with ~30% reductions in histone levels form apparently normal metaphase chromosomes, at least at the resolution of light microscopy (not shown). This indicates the reduction has more subtle effects. At the same time, we find that lower histone levels are not compensated for by merely increasing the nucleosomal repeat length to evenly distribute the remaining nucleosomes (not shown). Thus, one must assume there is a corresponding increase in non-nucleosomal DNA.

Chromatin structure plays a central role in the regulation of all DNA metabolic processes including transcription, replication, repair and recombination [61]. Post-translational modifications on the histone tails and histone variants are thought to be critical in the determination of cellular identity [90,91]. As every cell in a multi-cellular organism contains the same genomic information, cell differentiation and specialization require that distinct transcriptional programs are established and maintained [92,93]. Post-translational modifications on the histone tails are thought to impart cellular memory through epigenetic control of the genome [93]. Thus, every unique cell type is thought to exhibit a unique set of chromosome "marks", governing the genome-wide transcriptome and ultimately cellular identity. We suggest that a reduction of histone levels in HTLV-1 infected T-cells will result in loss of epigenetic information and may contribute to Tax deregulation of cellular gene expression leading to cancer.

We expect reduced histone levels to have similar effects as those observed with loss of imprinting (LOI) through DNA hypomethylation. These effects include aberrant chromosome rearrangements, deregulation of cellular gene expression and activation of latent viral genomes [94-100]. Reduction of histone levels could provide a means for HTLV-1 transcriptional reactivation after methylation of the 5' LTR in cultured ATLL cells [101].

Defects in chromatin assembly have been shown to impair all known pathways of double-strand break repair and activate S phase arrest [102,103]. Furthermore, partial depletion of histone H4 has been shown to increase homologous recombination-mediated genetic instability [104].

Chromatin structure, nucleosome remodeling and the epigenetic information encoded on the histone tails are known to participate in double-strand DNA break repair [105-108]. Defects in nucleosome assembly have been shown to interfere with the repair of double-strand DNA breaks [102]. We suggest that a Tax-mediated decrease in histone levels will result in an increase in the incidence of double-strand DNA breaks. The ability of Tax to increase the frequency of double-strand DNA break formation is consistent with its ability to increase the propensity for micronuclei formation [55,56]. Double-strand DNA breaks are potent inducers of genome instability as they are repaired by the error-prone process of non-homologous end joining, when a sister chromatid is not available for more efficient recombination-mediated repair [109]. Decreased histone levels may represent a previously unknown means for Tax induction of DNA damage.

## Conclusion

While the transforming capability of Tax has been demonstrated, the mechanisms of transformation by Tax are not completely understood. We suggest Tax repression of replication-dependent histone gene expression will result in reactivation of viral gene expression, deregulation of cellular gene expression and genomic instability. All of these effects may contribute to the development of adult T-cell leukemia/lymphoma. To our knowledge, this is the first example of a reduction of histone levels correlating with viral infection and cancer development.

## Methods

### Cell lines and cell culture

(C81, CEM, HuT102, Jurkat, Molt-4, and SLB-1) T-cell lines were cultured in Iscove's Modified Dulbecco's Media (IMDM; Sigma-Aldrich) supplemented with 10% fetal bovine serum, 50,000 units/liter each streptomycin/penicillin and 2 mM L-glutamine. All T-cell lines were cultured at 37°C in the presence of 5% CO_2_.

### RNA extraction and cDNA synthesis

RNA was extracted from asynchronous cells (10^7^) using Trizol (Invitrogen) in accordance with the manufacturer's protocol. In order to reduce genomic contamination, RNA samples were treated with 20 units of RNase-free DNase I (Roche) at room temperature for 30 minutes before heat inactivating the DNase I at 65°C for 10 minutes. cDNA was synthesized with the iScript cDNA synthesis kit (Bio-Rad) according to manufacturer's protocol using a programmable thermal cycler.

### Real-time PCR

Relative real-time PCR was performed using iQ SYBR Green Supermix (Bio-Rad) on an iCycler (Bio-Rad) and data expressed according to the Michael Pfaffl method, using EF1α as an internal control for normalization [110]. A five-point standard curve was constructed by serial diluting target DNA to evaluate PCR efficiency for every primer pair in every experiment. Reactions were performed in triplicate for every experiment. Real-time PCR amplicons were electrophoresed on 1.5% agarose gels to verify the specificity of each primer pair and melt curves were analyzed for every experiment to ensure that amplification products were primer pair specific. The following primer sequences were used for real-time PCR:

H2A Fwd AGCTCAACAAGCTTCTGGGCAA;

H2A Rev TTGTGGTGGCTCTCGGTCTTCTT;

H2B Fwd TGCGCCCAAGAAGGGTTCTAAA;

H2B Rev ACGAAGGAGTTCATGATGCCCA;

H3 Fwd TGCTCATCCGCAAACTGCCATT; H3 Rev AGTGACACGCTTGGCGTGAATA; H4 Fwd ACCGTAAAGTACTGCGCGACAA; H4 Rev TTCTCCAGGAACACCTTCAGCA; H1S-1 Fwd CCTGTAAAGAAGAAGGCGGCCAAA;

H1S-1 Rev CAGAGAAACTCCGCTACGCTCTTT;

H1S-2 Fwd CCCAGTATCTGAGCTTATCACCAAGG;

H1S-2 Rev TTTCTTAAGCGCGGCCAGAGAAAC;

H1S-3 Fwd CCCGGCTAAGAAGAAGGCAACTAA;

H1S-3 Rev GAAAGGCCATTGCGCTCCTTAGAA;

H1S-4 Fwd CCGGTGTCCGAGCTCATTACTAAA;

H1S-4 Rev GCTTTCTTGAGAGCGGCCAAAGAT;

EF1α Fwd GCCTCTCCAGGATGTCTACAAA;

EF1α Rev GTTTGAGAACACCAGTCTCCACTC;

Tax Fwd TTCTACCCGAAGACTGTTTGCCCA;

Tax Rev TGTCCAAATAAGGCCTGGAGTGGT.

### Histone content relative to DNA content determination by flow cytometry

Asynchronous cells (5 × 10^6^) from log phase culture were pelleted and suspended in ice-cold phosphate buffered saline (PBS). Cell suspensions were transferred to ice-cold 1% formaldehyde (methanol-free) in PBS and chilled on ice for 15 minutes to fix cells. Cells were washed with ice-cold PBS and transferred to ice-cold 70% ethanol and let stand at -20°C for 2 hours to permeablize cells. Cells were washed with PBS and incubated in 1% BSA-PBS containing 5 μg primary antibody (ab1791 rabbit polyclonal to Histone H3, AbCam or mouse monoclonal antibody directed against histone H1 product # V7013, Biomeda), overnight at 4°C while rotating. Cells were then washed with 1% BSA-PBS and incubated in a 1% BSA-PBS solution containing 5 μg of Alexa Fluor 488 goat anti-rabbit second antibody (product # A11008, Molecular Probes) for Histone H3 analysis or Alexa Fluor 488 goat anti-mouse second antibody (product # A11029, Molecular Probess) for Histone H1 analysis, for 1 hour at room temperature. Cells were washed with 1% BSA-PBS and incubated in PBS containing 25 ug/mL propidium iodide (Sigma) and 40 Kunitz U/mL RNase A (Sigma, certified DNase-free) for 30 minutes at room temperature. Cells were analyzed on a MoFlo (Dako Cytomation) flow cytometer/cell sorter with laser excitation at 488 nm with 110 mW. Data was interpreted using Summit software, version 4.0 (Dako Cytomation).

### Northern blots

Total RNA (10 μg) was resolved on 1% agarose/6.6% formaldehyde gels. Samples were transferred to a nylon membrane and probed with histone and EF1α probes simultaneously. PhosphorImager screens were scanned on a STORM (Molecular Probes). Histone signals were quantified with ImageQuant software version 5.1 (Molecular Dynamics) and normalized to EF1α signals. Probes were generated using a random primed DNA labeling kit (Roche). The DNA templates used for the generation of ^32^P-radiolabeled probes for histone and EF1α transcripts were prepared by PCR from Jurkat genomic DNA using the following primers:

H1 Fwd AAGGAGCGCAATGGCCTTTCTTTG;

H1 Rev GCCTTCTTGGCCTTTGCAGCTTTA;

H2A Fwd TTCAGTTTCCCGTAGGCCGAGT, H2A Rev GAAGCTTGTTGAGCTCCTCATCGT;

H2B Fwd AAGAAGGATGGCAAGAAGCGCAAG;

H2B Rev ACTTGGAGCTGGTGTACTTGGTGA;

H3 Fwd ATCGCTATCGGCCTGGTACAGT;

H3 Rev AGCTGGATGTCCTTGGGCATGATA;

H4 Fwd ATGTCAGGACGCGGCAAAGGAGGTAA;

H4 Rev GATCACGTTCTCCAGGAACACCTTCA;

EF1α Fwd TCATTGGACACGTAGATTCGGGCA;

EF1α Rev TTCACACCCAGTGTGTAAGCCAGA.

The DNA template used for the generation of ^32^P-radiolabeled probes for Tax transcripts was prepared by PCR from pSG-Tax plasmid DNA using the following primers:

Tax Fwd TTCCCAGGGTTTGGACAGAGTCTT;

Tax Rev GCGTGCCATCGGTAAATGTCCAAA.

### Co-transfection and magnetic selection of transfectants

Jurkat cells were co-transfected with pSG-Tax/pMACs K^k ^or pUC-19/pMACs K^k ^at 20 ug total DNA (3.5 molar excess of pMACs K^k^) per 10^7 ^cells by electroporation in 0.4 cm cuvettes using Genepulse (Bio-Rad). Transfected cells were enriched 24 hours following electroporation using the pMACS K^k ^magnetic selection system (Miltenyi Biotec) according to the manufacturer's protocol. Enriched cell populations were cultured for an additional 24 hours prior to harvesting cells for RNA extraction and DNA content analysis.

### DNA content analysis

Asynchronous cells (10^6^) from log phase culture were fixed with 70% ethanol and low molecular weight DNA was extracted with a 4 mM sodium citrate/192 mM sodium phosphate wash. Fixed cells were stained with 50 ug/mL propidium iodide in PBS containing 730 Kunitz U/mL RNase A for 20 minutes at room temperature. Data was collected on an EPICS flow cytometer (Beckman Coulter) and cell cycle distributions were calculated using Multicycle (Phoenix Flow Systems).

## Authors' contributions

JMB carried out the flow cytometry measurement of protein and DNA content, the reverse transcriptase/real-time PCR and Northern blot analyses of transcript levels in uninfected cells, HTLV-1 infected cells, mock transfected cells and Tax expression vector transfected cells. PJL conceived the study and participated in the design of the experiments and the interpretation of the results. All authors read and approved the final manuscript.
